# Preliminary Evaluation of Preoperative Optic Nerve Sheath Diameter and CT Mass Effect in Relation to Pre-Excision Invasive Intracranial Pressure During Intracranial Tumor Surgery

**DOI:** 10.3390/jcm15103807

**Published:** 2026-05-15

**Authors:** Khairunnisai Tarimah, Dewi Yulianti Bisri, Rohadi Muhammad Rosyidi, Elvan Wiyarta, Elya Endriani, Tatang Bisri

**Affiliations:** 1 Department of Anesthesiology and Intensive Therapy Subdivision Neuroanesthesia and Critical Care, Dr. Hasan Sadikin Hospital, Padjadjaran University, Bandung 40161, West Java, Indonesia; 2 Department of Anesthesiology and Intensive Therapy, Mataram Regional Hospital Kota Mataram, Al-Azhar Islamic University of Mataram, Mataram 83127, West Nusa Tenggara, Indonesia; 3 Department of Anesthesiology and Intensive Therapy Subdivision Neuroanesthesia and Critical Care, Jatinangor Hospital, Padjadjaran University, Bandung 40161, West Java, Indonesia; 4 Department of Neurosurgery, Medical Faculty, West Nusa Tenggara Province General Hospital, Mataram University, Mataram 83125, West Nusa Tenggara, Indonesia; 5 Intensive Care Department, University of Indonesia Hospital, Depok 16424, West Java, Indonesia; elvan.wiyarta@ui.ac.id; 6 Department of Anesthesiology and Intensive Therapy, Medical Faculty, Mataram University, West Nusa Tenggara Province General Hospital, Mataram 83125, West Nusa Tenggara, Indonesia; 7 Neuroanesthesia and Critical Care, Faculty of Jenderal Achmad Yani University, Cimahi 40531, West Java, Indonesia

**Keywords:** neoplasms, ultrasonography, tomography, craniotomy, monitoring

## Abstract

**Background:** Raised intracranial pressure in intracranial tumor surgery is driven by mass effect and edema, but invasive monitoring is selectively used, and imaging may not fully reflect contemporaneous pressure. We performed a pilot evaluation of preoperative optic nerve sheath diameter and CT mass effect in relation to pre-excision invasive intracranial pressure. **Methods:** This retrospective pilot study included adults with available preoperative optic nerve sheath diameter, CT mass effect graded by the Gordon–Firing score, and recorded pre-excision invasive intracranial pressure. The primary analysis assessed association with continuous pre-excision intracranial pressure using correlation and linear regression. Perioperative change in invasive intracranial pressure and serial optic nerve sheath diameter were also analyzed. Threshold analyses were exploratory. **Results:** In total, 45 patients were included. Mean pre-excision intracranial pressure was 29.40 mmHg, and 39/45 (86.7%) had intracranial pressure > 20 mmHg. Optic nerve sheath diameter showed a modest association with pre-excision intracranial pressure (r = 0.279, *p* = 0.064), whereas Gordon–Firing showed a stronger association (r = 0.522, *p* < 0.001). In the combined model, Gordon–Firing remained associated with intracranial pressure, whereas optic nerve sheath diameter did not. Mean intracranial pressure decreased by 12.24 mmHg after tumor excision, and optic nerve sheath diameter decreased at 1 h and 6 h postoperatively. **Conclusions:** CT mass effect graded by Gordon–Firing showed a stronger cross-sectional relationship with pre-excision invasive intracranial pressure than optic nerve sheath diameter, whereas serial optic nerve sheath diameter appeared more useful as a perioperative marker.

## 1. Introduction

Intracranial tumors can generate clinically important intracranial pressure burden through space-occupying mass effect, peritumoral edema, and disturbed cerebrospinal fluid dynamics, and extensive edema has been associated with worse early postoperative outcomes and higher complication rates in tumor surgery [1,2]. Reliable recognition of raised intracranial pressure remains important in perioperative neuro-oncology, but invasive monitoring is not universally feasible, and recent reviews and consensus documents continue to frame noninvasive intracranial pressure assessment as an unmet need and an adjunct rather than a replacement for invasive measurement [3,4,5].

Computed tomography remains the most accessible structural tool for preoperative assessment of intracranial mass effect. Early CT signs such as sulcal obliteration, ventricular compression, midline shift, and herniation correlate with measured intracranial pressure, but CT is still a static anatomical snapshot rather than a direct physiologic measurement [6]. Ultrasound measurement of optic nerve sheath diameter has therefore attracted considerable interest as a bedside surrogate because the optic nerve sheath is continuous with the intracranial subarachnoid space. Recent systematic and scoping reviews support ONSD as a promising noninvasive marker of raised intracranial pressure in both traumatic and nontraumatic brain injury, but they also show substantial heterogeneity in comparator standards and reported thresholds [7,8,9]. This heterogeneity is methodologically important because ONSD performance is sensitive to image acquisition and measurement quality, prompting the development of an international consensus quality criteria checklist for ONSD point-of-care ultrasonography [10].

More recent bedside studies continue to show positive correlation between ONSD and invasive intracranial pressure in critically ill neurological patients, while adjunct approaches that combine ONSD with additional ultrasound parameters are being explored to improve performance beyond ONSD alone [11,12]. In brain tumor surgery specifically, perioperative cohorts have shown that ONSD is often enlarged before surgery and decreases after tumor resection, supporting its responsiveness to perioperative intracranial dynamics [13,14].

However, in patients already selected for intracranial tumor surgery and often enriched for substantial mass effect, the clinically relevant question is not simply whether a single ONSD cutoff can classify intracranial hypertension, but whether ONSD tracks invasive pre-excision intracranial pressure and whether it adds physiologic information beyond routine structural imaging. Contemporary work in noninvasive intracranial pressure monitoring increasingly supports integrated assessment rather than reliance on a single bedside marker [3,4,5].

Accordingly, this study aimed to perform a preliminary pilot evaluation of preoperative ONSD and CT mass effect in relation to invasive pre-excision intracranial pressure in patients undergoing intracranial tumor surgery. We focused primarily on direct association with continuous invasive intracranial pressure and secondarily on exploratory threshold-based discrimination to assess whether ONSD provides additional information beyond CT mass effect in this high-risk perioperative setting.

## 2. Methods

### 2.1. Study Design, Setting, and Ethics

This was a single-center retrospective pilot observational study conducted at West Nusa Tenggara Regional Hospital using deidentified perioperative records of eligible patients managed between September and December 2025. Reporting was informed by STARD 2015 because the study included prespecified index tests and an invasive reference standard, although the primary analysis treated invasive ICP as a continuous outcome rather than a binary target [15]. Institutional review board approval was obtained from West Nusa Tenggara Regional Hospital under approval number 00.9/18/0479/RSUDP/2023, granted on 19 September 2023.

### 2.2. Participants

Adult patients who underwent intracranial tumor surgery with intraoperative subdural invasive ICP monitoring were eligible if preoperative ONSD measurement, preoperative CT for mass effect grading, and a recorded pre-excision invasive ICP value were available. Patients were excluded if any of these variables were missing. Consecutive eligible cases were identified from routine perioperative records.

### 2.3. Index Tests and Reference Standard

The first index test was preoperative ONSD measured by ocular ultrasonography in B mode. ONSD was measured 3 mm posterior to the globe in each eye, and the primary ONSD variable was the mean of both eyes. Retrospective reporting of ONSD acquisition was aligned as far as possible with current international quality criteria for ONSD imaging and measurement [10]. Operator-level metadata, such as probe frequency, operator training, image archiving, repeated caliper measurements, and formal inter-rater reliability assessment, were not consistently recoverable from the medical record and therefore were not incorporated into the analytic model. ONSD was assessed preoperatively, 1 h postoperatively, and 6 h postoperatively.

The second index test was CT mass effect graded using the Gordon–Firing score, an ordinal 0 to 4 scale defined as 0 for no mass effect, 1 for sulcal effacement, 2 for ipsilateral ventricular compression, 3 for midline shift, and 4 for contralateral ventricular dilatation [16]. A binary category was also prespecified as low mass effect for scores 0 to 1 and high mass effect for scores 2 to 4.

The reference standard was invasive ICP measured intraoperatively in mmHg using a subdural monitoring system placed as part of routine perioperative care. Two ICP values were extracted from the record, namely pre-excision ICP after monitor placement and stabilization but before tumor excision and post-excision ICP after tumor removal. The primary outcome was continuous pre-excision invasive ICP. Post-excision ICP was used only for paired perioperative change analysis. Device-level metadata, such as sensor architecture, zeroing or leveling method, external reference point, and waveform sampling frequency, were not consistently documented in the retrospective record and therefore could not be standardized across all cases. No undocumented monitor characteristics were assumed. Intracranial pressure was measured using a Camino intracranial pressure monitor (Integra LifeSciences, Princeton, NJ, USA). Ultrasonography was performed using a GE ultrasound system (GE HealthCare, Chicago, IL, USA).

### 2.4. Binary Thresholds

Because this cohort was expected to be enriched for elevated ICP, threshold-based analyses were prespecified as secondary and exploratory rather than primary. Three thresholds were examined, namely ICP greater than 20 mmHg, ICP greater than 22 mmHg, and ICP at least 25 mmHg. The threshold above 22 mmHg was included because the Brain Trauma Foundation guideline recommends treatment when ICP exceeds 22 mmHg [17]. The threshold above 20 mmHg was retained because it remains a commonly used definition of intracranial hypertension in the broader literature. The threshold of at least 25 mmHg was retained as a stricter exploratory threshold to permit comparison with the original analysis.

### 2.5. Statistical Analysis

Continuous variables were summarized as mean with standard deviation when approximately symmetric and as median with interquartile range when skewed. Categorical variables were summarized as counts and percentages. Two-sided *p*-values below 0.05 were considered statistically significant. All statistical analysis was done in Python (Python Software Foundation, USA).

The primary analysis examined direct association between each index test and continuous pre-excision invasive ICP using Pearson and Spearman correlation coefficients. Three prespecified linear models were then fitted with pre-excision invasive ICP as the dependent variable. Model 1 included ONSD alone. Model 2 included Gordon–Firing score alone. Model 3 included both variables. Model performance was summarized using regression coefficients, 95% confidence intervals, adjusted R^2^, Akaike information criterion, and leave-one-out cross-validated root mean squared error. Incremental information contributed by ONSD beyond CT mass effect was judged by change in R^2^, change in cross-validated root mean squared error, and partial R^2^ for ONSD within the combined model.

Perioperative change in invasive ICP from pre-excision to post-excision was analyzed using paired *t*-test and Wilcoxon signed-rank test. Serial ONSD across preoperative, 1 h postoperative, and 6 h postoperative time points were analyzed using a linear mixed effects model with time as a fixed effect and patient as a random intercept.

Secondary exploratory binary analyses were performed for ICP greater than 20 mmHg, greater than 22 mmHg, and at least 25 mmHg. For each threshold, three logistic models were fitted using ONSD alone, Gordon–Firing score alone, and both variables together. These exploratory models were summarized using apparent area under the receiver operating characteristic curve and leave-one-out cross-validated area under the curve. Bland–Altman analysis was not performed because ONSD and invasive ICP are different physiologic constructs measured on different scales and were not treated as interchangeable methods.

## 3. Results

**Figure 1:**
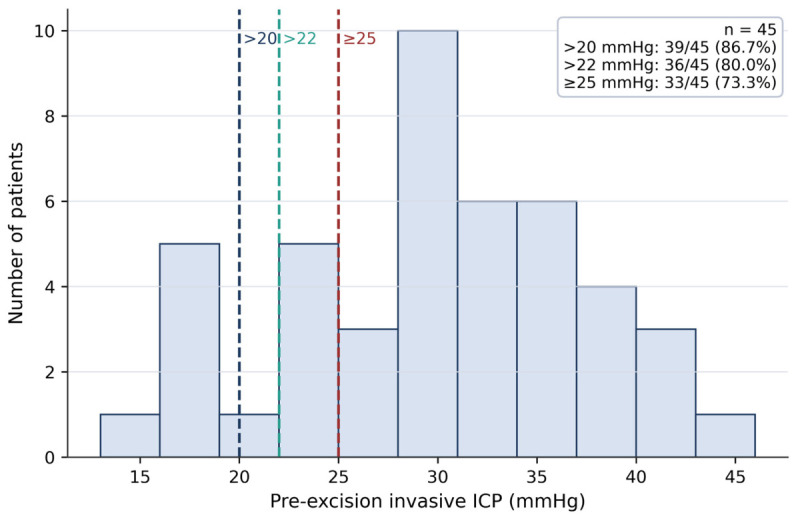
Distribution of pre-excision invasive intracranial pressure. Bars show the frequency distribution of patient-level pre-excision invasive ICP values. ICP: intracranial pressure.

A total of 45 patients with complete preoperative ONSD, CT mass effect grading, and paired invasive ICP measurements were included. Mean age was 47.4 years, and 32 patients were female, representing 71.1% of the cohort. The most common tumor diagnosis or category was meningioma in 27 patients (60.0%), followed by glioblastoma in 9 patients (20.0%). Tumors were right-sided in 22 patients (48.9%), left-sided in 16 patients (35.6%), and midline in 7 patients (15.6%). Most tumors were supratentorial extra axial in 27 patients (60.0%), followed by supratentorial intra axial in 16 patients (35.6%) and infratentorial extra axial in 2 patients (4.4%). Mean pre-excision invasive ICP was 29.40 mmHg with an SD of 7.49, while the median was 29.0 mmHg with an IQR of 24.0 to 35.0. Mean post-excision invasive ICP was 17.16 mmHg with an SD of 5.67. Mean preoperative ONSD was 5.40 mm with an SD of 0.67, decreasing to 4.50 mm at 1 h postoperatively and 4.68 mm at 6 h postoperatively. The median Gordon–Firing score was 2.0 with an IQR of 1.0 to 3.0. Elevated ICP was common, with 39 of 45 patients, or 86.7%, exceeding 20 mmHg; 36 of 45, or 80.0%, exceeding 22 mmHg; and 33 of 45, or 73.3%, reaching at least 25 mmHg. Baseline cohort characteristics are summarized in Table 1, and the distribution of pre-excision invasive ICP is shown in Figure 1.

**Figure 2:**
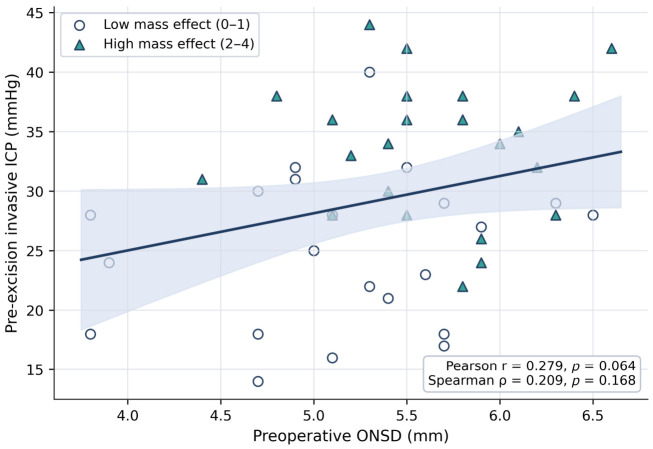
Relationship between preoperative ONSD and pre-excision invasive ICP. Points represent individual patients and are colored according to CT mass effect category. The solid line indicates the fitted linear regression line, and the shaded band indicates the 95% confidence interval of the fitted mean. ICP: intracranial pressure; ONSD: optic nerve sheath diameter.

Direct association analyses showed that preoperative ONSD had only a modest relationship with pre-excision invasive ICP. The Pearson correlation between ONSD and ICP was 0.279 with a *p*-value of 0.064, while the Spearman correlation was 0.209 with a *p*-value of 0.168. In contrast, CT mass effect showed a stronger association with invasive ICP. Gordon–Firing score correlated with pre-excision ICP with a Pearson correlation of 0.522 and a Spearman correlation of 0.508, both with *p*-values below 0.001. These direct associations, together with the scatter distribution across CT mass effect strata, are presented in Table 2 and Figure 2.

In continuous linear models, ONSD alone showed a regression coefficient of 3.13 with a 95% CI of −0.19 to 6.44 and a *p*-value of 0.064, with an adjusted R^2^ of 0.056, AIC of 308.3, and LOOCV RMSE of 7.40. Gordon–Firing score alone performed better, with a regression coefficient of 2.66, a 95% CI of 1.33 to 4.00, and a *p*-value below 0.001, with an adjusted R^2^ of 0.256, AIC of 297.6, and LOOCV RMSE of 6.59. In the combined model, Gordon–Firing score remained independently associated with invasive ICP, with a coefficient of 2.46, a 95% CI of 1.10 to 3.83, and a *p*-value below 0.001, whereas ONSD was no longer independently associated after adjustment, with a coefficient of 1.86, a 95% CI of −1.15 to 4.87, and a *p*-value of 0.219. The combined model yielded an adjusted R^2^ of 0.266, AIC of 298.0, and LOOCV RMSE of 6.57. Adding ONSD on top of Gordon–Firing produced only a small increment in model performance, with ΔR^2^ of 0.026, partial R^2^ of 0.036, and ΔLOOCV RMSE of −0.02. These model results are presented in Table 2.

**Figure 3:**
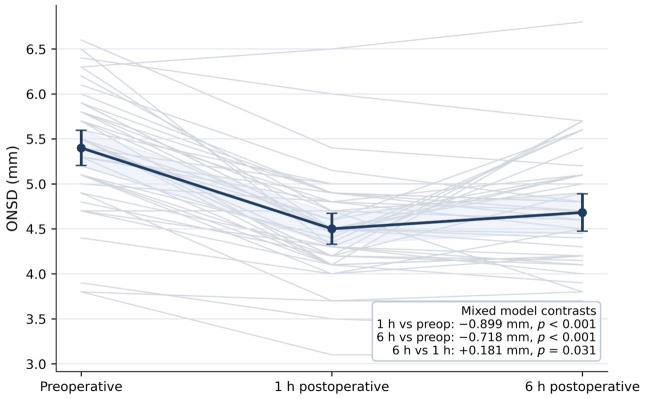
Serial optic nerve sheath diameter across perioperative time points. Thin lines represent individual patient trajectories from preoperative assessment to 1 h and 6 h postoperatively. The thicker line represents the mean profile across time points. ONSD: optic nerve sheath diameter.

**Figure 4:**
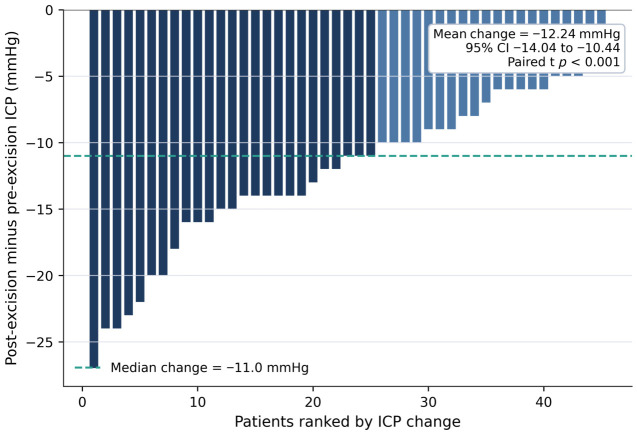
Patient-level change in invasive ICP after tumor excision. Bars represent patient-level ICP change calculated as post-excision minus pre-excision ICP and are sorted by magnitude of change. Negative values indicate lower ICP after tumor excision. ICP: intracranial pressure.

Paired perioperative analysis showed a marked reduction in invasive ICP after tumor excision. Mean ICP change from pre-excision to post-excision was −12.24 mmHg with a 95% CI of −14.04 to −10.44, and both paired *t*-test and Wilcoxon signed-rank test showed *p*-values below 0.001. Serial ONSD measurements also decreased after surgery. Compared with the preoperative value, ONSD was lower at 1 h postoperatively by 0.899 mm with a 95% CI of −1.064 to −0.734 and a *p*-value below 0.001 and lower at 6 h postoperatively by 0.718 mm with a 95% CI of −0.883 to −0.553 and a *p*-value below 0.001. A small rebound was observed between 1 h and 6 h, with a mean increase of 0.181 mm, a 95% CI of 0.016 to 0.346, and a *p*-value of 0.031. These perioperative changes are summarized in Table 3 and illustrated in Figure 3 and Figure 4.

Exploratory threshold-based analyses showed only modest discrimination after internal validation. For ICP above 20 mmHg, apparent AUCs were 0.716 for ONSD, 0.667 for Gordon–Firing, and 0.791 for the combined model, whereas LOOCV AUCs were 0.594, 0.590, and 0.684, respectively. For ICP above 22 mmHg, apparent AUCs were 0.647, 0.653, and 0.707, with corresponding LOOCV AUCs of 0.500, 0.596, and 0.596. For ICP at least 25 mmHg, apparent AUCs were 0.631, 0.679, and 0.736, with corresponding LOOCV AUCs of 0.523, 0.624, and 0.654. Given the high prevalence of elevated ICP in this cohort, these threshold-based analyses were treated as exploratory and are presented in Supplementary Table S1 and Figure S1.

## 4. Discussion

The main finding of this pilot study is that preoperative ONSD showed only a modest association with pre-excision invasive ICP, whereas CT mass effect graded by Gordon–Firing showed a stronger relationship with invasive pressure and remained associated with ICP in the combined model. The additional contribution of ONSD beyond CT was small, and the exploratory threshold-based analyses remained only modest after internal validation. By contrast, serial ONSD decreased clearly after tumor excision and paralleled the marked fall in invasive ICP, suggesting that in intracranial tumor surgery, ONSD may be more informative as a dynamic perioperative physiologic marker than as a strong standalone cross-sectional classifier of intracranial hypertension.

The stronger signal observed for Gordon–Firing is biologically plausible. In a cohort already selected for intracranial tumor surgery, structural derangements such as sulcal effacement, ventricular compression, and midline shift likely account for a large proportion of the pre-excision pressure burden. Contemporary reviews of ICP monitoring increasingly emphasize that ICP should not be interpreted only through a single threshold, but within a broader framework of compliance, intracranial dynamics, and ancillary information [18,19]. CT-based signs of mass effect have been shown to correlate with measured ICP, but CT remains an anatomical snapshot rather than a direct physiologic measurement [6]. This helps explain why CT mass effect in our data carried more cross-sectional signal than ONSD, yet still should not be interpreted as a substitute for direct pressure measurement [20].

The modest cross-sectional performance of ONSD in our study is also consistent with the broader literature. Recent reviews continue to support ONSD as a useful noninvasive marker of raised ICP, but they repeatedly emphasize heterogeneity in thresholds, reference standards, and study populations [21,22]. This heterogeneity is clinically important because ONSD is highly dependent on image acquisition and measurement quality, which led to development of the international ONSD POCUS quality criteria checklist [10]. More recent bedside data still support a positive correlation between ONSD and ICP in neurologically ill patients, but they also reinforce that performance depends heavily on context and technique rather than on a universally portable cutoff [11].

Our perioperative findings support the physiologic responsiveness of ONSD. Prior perioperative tumor cohorts have shown that ONSD changes across operative time points and is influenced by tumor characteristics and clinical context [14]. Recent longitudinal work also suggests that intraindividual ONSD trajectories may correlate with ICP more meaningfully than a single cross-sectional value, raising the possibility that personalized follow-up may be more informative than a one-size-fits-all threshold [23]. This interpretation is also consistent with emerging work on automated and real-time ONSD measurement, which is aimed less at defending one universal cutoff and more at improving repeated bedside monitoring [12].

The exploratory threshold-based results should therefore be interpreted cautiously. This cohort was highly enriched for elevated ICP, especially at the lower thresholds, so binary discrimination was inherently constrained by a narrow clinical spectrum and a small number of low-pressure cases. Recent expert reviews and surveys suggest that when ICP is in the borderline or intermediate range, many clinicians already consider ancillary physiologic information before escalating treatment, rather than relying on a single threshold in isolation [19,24]. In that context, the modest apparent AUC values in our study and their further attenuation after internal validation are not surprising, and they argue against overinterpreting the threshold-based analyses as clinically actionable diagnostic performance estimates.

Our findings are more aligned with a multimodal interpretation strategy. Recent reviews and consensus documents increasingly frame noninvasive ICP assessment as an integrated bedside process that combines examination, CT, and ultrasound rather than a search for a single replacement test [5,20,21]. Within that framework, our data suggest that Gordon–Firing captures the dominant structural signal before decompression, whereas ONSD contributes a smaller adjunctive signal and may be more useful for tracking change over time.

This study also has several strengths. It used recorded invasive ICP rather than a purely imaging-based surrogate, allowing direct comparison of ONSD and CT mass effect against a clinically relevant reference standard. The analysis was deliberately centered on continuous physiologic association rather than on threshold-based classification alone, which is more appropriate for a cohort already enriched for elevated ICP. In addition, paired pre- and post-excision invasive ICP measurements together with serial ONSD measurements provided a perioperative dataset that is uncommon in routine tumor surgery practice.

The limitations are important and should shape interpretation. First, this was a retrospective single-center study with a small sample, so precision and transportability are limited. Tumor volume was not consistently available in the retrospective dataset and therefore could not be incorporated into the baseline table or analytic models. Because tumor burden may influence mass effect, postoperative edema, and intracranial pressure dynamics, future prospective studies should include standardized volumetric tumor assessment. Second, the reference standard was based on clinically recorded subdural invasive ICP values, but device-level metadata such as sensor architecture, zeroing method, reference point, and sampling frequency were not consistently available. A recent pilot validation study supports feasibility of subdural monitoring and showed strong correlation with intraventricular ICP, but site-related differences remain relevant near decision thresholds [25]. Third, operator-level metadata for ONSD acquisition, including probe frequency, training, repeated caliper measurements, and formal inter-rater reliability, were not consistently recoverable from the record [10]. Fourth, perioperative ONSD may be influenced by factors beyond pressure alone. In adults undergoing supratentorial tumor craniotomy, ONSD behavior has been shown to vary with perioperative ventilatory conditions, indicating that the sonographic signal should be interpreted within the broader anesthetic and physiologic context [26]. Finally, because the cohort was highly enriched for elevated ICP, the threshold-based analyses should be regarded as exploratory only and not as practice-changing diagnostic accuracy estimates.

Taken together, these findings support a restrained clinical interpretation. In patients undergoing intracranial tumor surgery, preoperative ONSD alone should not be considered a robust standalone surrogate for pre-excision invasive ICP. CT mass effect carries stronger cross-sectional information in this setting, while ONSD appears more useful as an adjunct marker that may contextualize structural imaging and reflect perioperative pressure dynamics after decompression. Future work should prioritize prospective multicenter tumor cohorts with standardized ONSD acquisition, explicit invasive monitor metadata, and predefined analyses centered on continuous ICP rather than threshold hunting alone.

## 5. Conclusions

In this pilot study of intracranial tumor surgery, preoperative optic nerve sheath diameter showed only a modest association with pre-excision invasive intracranial pressure, while CT mass effect graded by Gordon–Firing demonstrated a stronger cross-sectional relationship. The additional contribution of optic nerve sheath diameter beyond CT was small, but serial postoperative measurements paralleled the decline in invasive intracranial pressure after tumor excision, supporting its value as a dynamic perioperative marker rather than a robust standalone surrogate. These findings support a restrained multimodal interpretation in which optic nerve sheath diameter complements, but does not replace, structural imaging and invasive pressure assessment. Prospective multicenter studies with standardized optic nerve sheath diameter acquisition and more completely characterized invasive monitoring are needed before broader clinical adoption.

## Figures and Tables

**Table 1:** Baseline cohort characteristics and invasive ICP distribution.

Characteristic	Value
*N*	45
Age, mean (SD)	47.4 (13.2)
Female, *n* (%)	32 (71.1%)
Tumour diagnosis or category, *n* (%)	
Meningioma	27 (60.0%)
Glioblastoma	9 (20.0%)
Pineal region lesion, type not specified	2 (4.4%)
Other CNS tumour, type not specified	2 (4.4%)
Pituitary neuroendocrine tumour	2 (4.4%)
Metastatic tumour to the brain	1 (2.2%)
Cerebellopontine angle tumour, type not specified	1 (2.2%)
Diffuse glioma, adult type, non-glioblastoma	1 (2.2%)
Tumour side, *n* (%)	
Right	22 (48.9%)
Left	16 (35.6%)
Midline	7 (15.6%)
Tumour location category, *n* (%)	
Supratentorial extra axial	27 (60.0%)
Supratentorial intra axial	16 (35.6%)
Infratentorial extra axial	2 (4.4%)
Pre-excision ICP, mean (SD)	29.40 (7.49)
Pre-excision ICP, median [IQR]	29.0 [24.0, 35.0]
Post-excision ICP, mean (SD)	17.16 (5.67)
Preoperative ONSD, mean (SD)	5.40 (0.67)
1 h postoperative ONSD, mean (SD)	4.50 (0.59)
6 h postoperative ONSD, mean (SD)	4.68 (0.71)
Gordon–Firing score, median [IQR]	2.0 [1.0, 3.0]
ICP > 20 mmHg, *n* (%)	39 (86.7%)
ICP > 22 mmHg, *n* (%)	36 (80.0%)
ICP ≥ 25 mmHg, *n* (%)	33 (73.3%)

Values are presented as mean ± standard deviation, median [interquartile range], or *n*/*N* (%). ICP: intracranial pressure; IQR: interquartile range; ONSD: optic nerve sheath diameter; SD: standard deviation.

**Table 2:** Direct associations and continuous linear models for pre-excision invasive ICP.

Panel	Analysis	Estimate	95% CI	*p*-Value	Model Fit
Direct association	ONSD vs. pre-excision ICP	Pearson r = 0.279	-	*p* = 0.064	-
Direct association	ONSD vs. pre-excision ICP	Spearman rho = 0.209	-	*p* = 0.168	-
Direct association	Gordon–Firing vs. pre-excision ICP	Pearson r = 0.522	-	*p* < 0.001	-
Direct association	Gordon–Firing vs. pre-excision ICP	Spearman rho = 0.508	-	*p* < 0.001	-
Linear model	Model 1: ONSD-ONSD	Beta = 3.13	−0.19 to 6.44	*p* = 0.064	Adj R^2^ = 0.056; AIC = 308.3; LOOCV RMSE = 7.40
Linear model	Model 2: Gordon–Firing-Gordon–Firing	Beta = 2.66	1.33 to 4.00	*p* < 0.001	Adj R^2^ = 0.256; AIC = 297.6; LOOCV RMSE = 6.59
Linear model	Model 3: ONSD + Gordon–Firing-ONSD	Beta = 1.86	−1.15 to 4.87	*p* = 0.219	Adj R^2^ = 0.266; AIC = 298.0; LOOCV RMSE = 6.57
Linear model	Model 3: ONSD + Gordon–Firing-Gordon–Firing	Beta = 2.46	1.10 to 3.83	*p* < 0.001	Adj R^2^ = 0.266; AIC = 298.0; LOOCV RMSE = 6.57
Incremental information	Added ONSD on top of Gordon–Firing	ΔR^2^ = 0.026	Partial R^2^ = 0.036	*p* = 0.219	ΔLOOCV RMSE = −0.02

Pearson and Spearman statistics summarize direct association with continuous ICP. Linear model rows report predictor-specific coefficients within each model. LOOCV indicates leave-one-out cross-validation. 95% CI and model fit are not applicable to correlation coefficients. ΔLOOCV RMSE is relative to the Gordon–Firing only model.

**Table 3:** Perioperative change in invasive ICP and serial ONSD.

Domain	Contrast	Estimate	95% CI	*p*-Value
Invasive ICP	Post-excision minus pre-excision	−12.24 mmHg	−14.04 to −10.44	Paired *t p* < 0.001; Wilcoxon *p* < 0.001
Serial ONSD	1 h postoperative vs. preoperative	−0.899 mm	−1.064 to −0.734	*p* < 0.001
Serial ONSD	6 h postoperative vs. preoperative	−0.718 mm	−0.883 to −0.553	*p* < 0.001
Serial ONSD	6 h postoperative vs. 1 h postoperative	0.181 mm	0.016 to 0.346	*p* = 0.031

The ICP contrast is based on paired within-patient change. ONSD contrasts are from a linear mixed model with a random patient intercept.

## Data Availability

All data generated or analyzed during the study are included in this published article.

## Supplementary Materials

The following supporting information can be downloaded at: https://www.mdpi.com/article/10.3390/jcm15103807/s1, Supplementary Table S1: Exploratory binary threshold model performance; Supplementary Figure S1: Exploratory ROC curves across binary ICP thresholds. AUC: area under the curve; ICP: intracranial pressure; ONSD: optic nerve sheath diameter; ROC: receiver operating characteristic.
