# Supplementary material for: Preliminary Evaluation of Preoperative Optic Nerve Sheath Diameter and CT Mass Effect in Relation to Pre-Excision Invasive Intracranial Pressure During Intracranial Tumor Surgery
Source: J Clin Med. 2026 May 15;15(10):3807. doi: 10.3390/jcm15103807 (PMC13208005; doi:10.3390/jcm15103807)

Supplementary Materials

Supplementary Table S1. Exploratory binary threshold model performance

Threshold	Model	Apparent AUC	LOOCV AUC	LOOCV Brier
ICP >20 mmHg	ONSD	0.716	0.594	0.124
ICP >20 mmHg	Gordon-Firing	0.667	0.590	0.122
ICP >20 mmHg	Combined model	0.791	0.684	0.128
ICP >22 mmHg	ONSD	0.647	0.500	0.172
ICP >22 mmHg	Gordon-Firing	0.653	0.596	0.167
ICP >22 mmHg	Combined model	0.707	0.596	0.174
ICP ≥25 mmHg	ONSD	0.631	0.523	0.203
ICP ≥25 mmHg	Gordon-Firing	0.679	0.624	0.199
ICP ≥25 mmHg	Combined model	0.736	0.654	0.201

These analyses are exploratory because the cohort is highly enriched for elevated ICP, especially at lower thresholds.

Supplementary Figure S1. Exploratory ROC curves across binary ICP thresholds. AUC: area under the curve; ICP: intracranial pressure; ONSD: optic nerve sheath diameter; ROC: receiver operating characteristic.

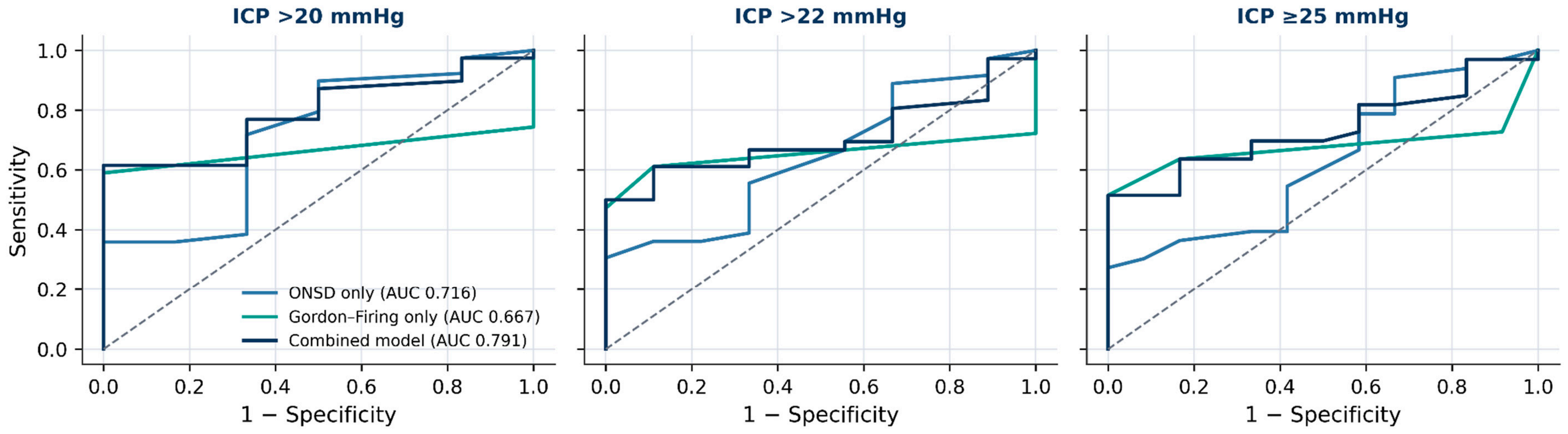

Supplement: Supplementary file 1 [file jcm-15-03807-s001.zip › jcm-4298269-supplementary.pdf]
